# Dental Fees

**Published:** 1900-01

**Authors:** 


ARTICLE V.

### DENTAL FEES.

Perhaps there is no class of professional men so much abused by the public generally for charging exorbitant prices as dentists, and it is certain that many times they are unjustly accused.

In discussing this question, I propose to do so quite briefly, and from a single standpoint, but not in any sort as an apologist. I come to claim boldly and unequivocably for the competent dentist a liberal remuneration, and it is believed that facts can be stated, appealing to the sense of justice of most individuals, which shall establish the claim of such dentists to the most generous compensation.

It must be remembered that the qualified dentist spends three or four years in preparatory study, and generally many years in establishing a reputation that shall bring him a remunerative practice. When this is accomplished, he finds himself embarked in one of the most trying and unhealthy occupations he could have selected.

The operative dentist is confined almost exclusively to his office, scarcely during the day getting a breath of pure, fresh air, while he is forced frequently to breathe an atmosphere absolutely contaminated, and that to a degree that must be experienced to be appreciated. He is constantly kept in a position the most tiresome and unhealthy, while the character of his labors is extremely exhaustive. Thus having been deprived of fresh air to breathe, of all healthful or invigorating exercise, the close of each day finds him wearied and prostrated, mentally as well as bodily. Still his labor for the day is not yet done. He has had no time during the day for reading or study, and must rob himself of needed recreation of the evening, or else of some portion of his equally needed sleep and rest, that this very important part of his duty to himself and his parents may not be neglected. Is it not clear, then, that the occupation of the dentist is necessarily an unhealthy one? If this is so, you will be surprised at the statement that very few dentists can continue in full practice for many years, without finding their health seriously impaired, or utterly broken.

It may safely be said that nine out of every ten dentists who have attained to anything like success or competence by means of their profession alone, have done so at the expense of their health. Either they have been unable to follow longer the practice of their profession, or they have followed it to so limited an extent that their income from it has proved but normal and very uncertain.

Now upon this statement of facts—and they are facts—is based the claim which has been asserted.

Not taking into consideration the high order of skill requisite in the dentist, a skill, which, when attained, must always command a high price, nor the fact of his almost entire exclusion from all out-of door enjoyments and recreations, but simply upon the ground of the absolutely unhealthy character of his occupation, and the almost certainty of the loss of health in obtaining a competence, is asserted of the skilled and competent dentist to a glorious reward.

Clearly, the dentist can only make provision for the future while his health and strength enable him to work, before those dark days come—which are so sure to come—days when he can no longer labor, and when perhaps his life will be a continual struggle with disease, engendered by his devotion to his profession. In a word, he must condense within the compass of a few years the labor and results of a lifetime.

I think I have made good the claim I have advanced, but if I have not at all, to those who are disposed to deny the claim, I have only to say, I wish they might have the personal experience, and they would accord all that is demanded in this article.

And now, kind reader, one word as to what prompted this article, and I have done. I know that the public, generally, do not understand this matter in its true light, nor could they be expected to. I know, too, that skilled dentists of established reputations will continue to charge high fees, and the public will continue to pay them—but I hoped in writing this article—basing the claim which I have endeavored to substantiate, upon what seemed to me to be its true and legitimate grounds—to establish a more kindly feeling between patients and their dentists than perhaps would otherwise have existed. I hoped, too, to induce some of my readers to believe that all dentists are not actuated by simply sordid motives. There are, perhaps, as many of the dental as of the medical profession who are impelled by high and noble motives, laboring assiduously and conscientiously for the alleviation of suffering and for the highest interests of their patients.

Asking you to give this subject a fair and candid consideration, let me believe that I have not wholly failed in my undertaking.—*Information.*

